# 2,3-Dibromo-1,3-bis­(4-fluoro­phen­yl)propan-1-one

**DOI:** 10.1107/S1600536810026905

**Published:** 2010-07-14

**Authors:** Jerry P. Jasinski, Curtis J Guild, S. Samshuddin, B. Narayana, H. S. Yathirajan

**Affiliations:** a Department of Chemistry, Keene State College, 229 Main Street, Keene, NH 03435-2001, USA; b Department of Studies in Chemistry, Mangalore University, Mangalagangotri 574 199, India; c Department of Studies in Chemistry, University of Mysore, Manasagangotri, Mysore 570 006, India

## Abstract

In the title compound, C_15_H_10_Br_2_F_2_O, the dihedral angle between the two 3-fluoro-substituted benzene rings is 5.7 (5)°. The two bromine substituents on the chalcone moiety are close to *anti* as the Br—C—C—Br torsion angle is 176.9 (7)°. Weak C—Br⋯π inter­actions may contribute to the crystal stability.

## Related literature

For bromo substitution of non-linerar optical (NLO) compounds, see: Uchida *et al.* (1998 ▶); Tam *et al.* (1989 ▶); Indira *et al.* (2002 ▶). For NLO first-order hyperpolarizabilities, see: Zhao *et al.* (2002 ▶). For related structures, see: Narayana *et al.* (2007 ▶); Sarojini *et al.* (2007 ▶); Yathirajan *et al.* (2007 ▶); Butcher *et al.* (2006 ▶).
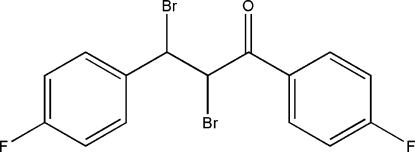


## Experimental

### 

#### Crystal data

C_15_H_10_Br_2_F_2_O
*M* *_r_* = 404.05
Triclinic, 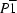

*a* = 5.7381 (13) Å
*b* = 9.909 (2) Å
*c* = 12.575 (3) Å
α = 75.324 (3)°
β = 87.472 (3)°
γ = 82.300 (3)°
*V* = 685.4 (3) Å^3^
*Z* = 2
Mo *K*α radiation
μ = 5.93 mm^−1^
*T* = 100 K
0.55 × 0.30 × 0.25 mm

#### Data collection

Bruker APEXII CCD diffractometer
Absorption correction: multi-scan (*SADABS*; Bruker, 2008 ▶) *T*_min_ = 0.329, *T*_max_ = 0.746
8841 measured reflections
4008 independent reflections
3408 reflections with *I* > 2σ(*I*)
*R*_int_ = 0.032

#### Refinement

*R*[*F* ^2^ > 2σ(*F* ^2^)] = 0.031
*wR*(*F* ^2^) = 0.079
*S* = 1.21
4008 reflections
181 parameters
H-atom parameters constrained
Δρ_max_ = 1.08 e Å^−3^
Δρ_min_ = −0.87 e Å^−3^

### 

Data collection: *APEX2* (Bruker, 2008 ▶); cell refinement: *SAINT* (Bruker, 2008 ▶); data reduction: *SAINT*; program(s) used to solve structure: *SHELXTL* (Sheldrick, 2008 ▶); program(s) used to refine structure: *SHELXTL*; molecular graphics: *SHELXTL*; software used to prepare material for publication: *SHELXTL*.

## Supplementary Material

Crystal structure: contains datablocks global, I. DOI: 10.1107/S1600536810026905/tk2690sup1.cif

Structure factors: contains datablocks I. DOI: 10.1107/S1600536810026905/tk2690Isup2.hkl

Additional supplementary materials:  crystallographic information; 3D view; checkCIF report

## Figures and Tables

**Table 1:** *Y*—*X*⋯*Cg* inter­actions (Å) *Cg*1 and *Cg*2 are the centroids of the C1–C6 and C10–C15 rings, respectively.

*Y*—*X*⋯*Cg*	*X*⋯*Cg*	*Y*⋯*Cg*	*Y*—*X*⋯*Cg*
C8—Br1⋯*Cg*1^i^	3.650 (7)	5.617 (2)	174
C7—Br3⋯*Cg*2^ii^	3.479 (6)	5.341 (1)	153

Symmetry codes: (i) −*x*, 1 − *y*, 1 − *z*; (ii) 1 − *x*, 1 − *y*, −*z*.

**Fig. 1.**
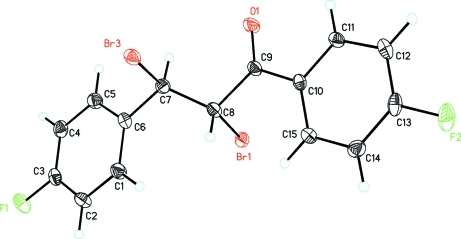
Molecular structure of C15H10F2Br2O, showing the atom labeling scheme and 50% probability displacement ellipsoids.

## supplementary crystallographic information

### Comment

The non-linear optical (NLO) effect in organic molecules originates from a strong donor–acceptor intermolecular interaction, a delocalized π-electron system and the ability to crystallize in a non-centrosymmetric space group. Among several organic compounds exhibiting NLO effects, chalcone derivatives are important materials known for their excellent blue light transmittance and good crystallizability. It has been observed that substitution of a bromo group on either of the phenyl rings greatly influences non-centrosymmetric crystal packing (Uchida *et al.*, 1998; Tam *et al.*, 1989; Indira *et al.*, 2002). Bromo substituents can obviously improve molecular first-order hyperpolarizabilities and can effectively reduce dipole–dipole interactions between molecules (Zhao *et al.*, 2002). Chalcone derivatives usually have lower melting points, which can be a drawback when their crystals are used in optical instruments. Chalcone dibromides usually have higher melting points and are thermally stable.

The crystal structures of some dibromo chalcones *viz*., 2,3-dibromo- 3-(5-bromo-2-methoxyphenyl)-1-(2,4-dichlorophenyl)propan-1-one (Narayana *et al.*, 2007), 2,3-dibromo-3-(4-bromo-6-methoxy-2 -naphthyl)-1-(4-methoxyphenyl)propan-1-one (Sarojini *et al.*, 2007), 2,3-dibromo-1-(3-bromo-2-thienyl)-3-(4-fluorophenyl)propan-1-one, (Yathirajan *et al.*, 2007), 2,3-dibromo-1-(4-methoxyphenyl)-3-[4- (methylsulfanyl)phenyl] propan-1-one, (Butcher *et al.*, 2006) have been reported. In continuation of our studies on chalcones and their derivatives, the title chalcone dibromide, C_15_H_10_F_2_Br_2_O, was prepared by the bromination of the chalcone precursor, and its crystal structure is reported.

The title compound, C_15_H_10_F_2_Br_2_O, contains two *m*-fluoro-substituted rings attached to a brominated chalcone moiety. The dihedral angle between the mean planes of the benzene rings is 5.7 (5) °. The two bromine substituents on the chalcone moiety are nearly opposite to each orher [Br1–C8–C7–Br2 = 176.9 (7) °]. Weak C—Br···π interactions (Table 1) contribute to crystal stability.

### Experimental

To a solution of (2*E*)-1,3-bis(4-fluorophenyl)prop-2-en-1-one (2.44 g, 0.01 mol) in acetic acid (25 ml), bromine (1.60 g, 0.01 mol) in acetic acid (10 ml) was added slowly with stirring at 273 K. After completion of the addition of the bromine solution, the reaction mixture was stirred for 5 h. The solid obtained was filtered and recrystallized from acetone. The crystals were grown from methanol by slow evaporation and the yield of the compound was 86%. (m.pt. 443 K). Analytical data: Found (Calculated): C %: 44.57 (44.59); H%: 2.48 (2.49).

### Refinement

All of the H atoms were placed in their calculated positions and then refined using the riding model with C—H = 0.93–0.98 Å, and with *U*_iso_(H) = 1.17–1.23*U*_eq_(C).

### Crystal data

**Table:**

C_15_H_10_Br_2_F_2_O	*Z* = 2
*M**_r_* = 404.05	*F*(000) = 392
Triclinic, *P*1	*D*_x_ = 1.958 Mg m^−^^3^
Hall symbol: -P 1	Melting point: 443 K
*a* = 5.7381 (13) Å	Mo *K*α radiation, λ = 0.71073 Å
*b* = 9.909 (2) Å	Cell parameters from 3859 reflections
*c* = 12.575 (3) Å	θ = 2.4–31.2°
α = 75.324 (3)°	µ = 5.93 mm^−^^1^
β = 87.472 (3)°	*T* = 100 K
γ = 82.300 (3)°	Block, colourless
*V* = 685.4 (3) Å^3^	0.55 × 0.30 × 0.25 mm

### Data collection

**Table:**

Bruker APEXII CCD diffractometer	4008 independent reflections
Radiation source: fine-focus sealed tube	3408 reflections with *I* > 2σ(*I*)
graphite	*R*_int_ = 0.032
ω scans	θ_max_ = 31.1°, θ_min_ = 2.1°
Absorption correction: multi-scan (*SADABS*; Bruker, 2008)	*h* = −8→8
*T*_min_ = 0.329, *T*_max_ = 0.746	*k* = −14→14
8841 measured reflections	*l* = −17→17

### Refinement

**Table:**

Refinement on *F*^2^	Primary atom site location: structure-invariant direct methods
Least-squares matrix: full	Secondary atom site location: difference Fourier map
*R*[*F*^2^ > 2σ(*F*^2^)] = 0.031	Hydrogen site location: inferred from neighbouring sites
*wR*(*F*^2^) = 0.079	H-atom parameters constrained
*S* = 1.21	*w* = 1/[σ^2^(*F*_o_^2^) + (0.0334*P*)^2^] where *P* = (*F*_o_^2^ + 2*F*_c_^2^)/3
4008 reflections	(Δ/σ)_max_ = 0.001
181 parameters	Δρ_max_ = 1.08 e Å^−^^3^
0 restraints	Δρ_min_ = −0.87 e Å^−^^3^

### Special details

**Table:**

Geometry. All e.s.d.'s (except the e.s.d. in the dihedral angle between two l.s. planes) are estimated using the full covariance matrix. The cell e.s.d.'s are taken into account individually in the estimation of e.s.d.'s in distances, angles and torsion angles; correlations between e.s.d.'s in cell parameters are only used when they are defined by crystal symmetry. An approximate (isotropic) treatment of cell e.s.d.'s is used for estimating e.s.d.'s involving l.s. planes.
Refinement. Refinement of *F*^2^ against ALL reflections. The weighted *R*-factor *wR* and goodness of fit *S* are based on *F*^2^, conventional *R*-factors *R* are based on *F*, with *F* set to zero for negative *F*^2^. The threshold expression of *F*^2^ > σ(*F*^2^) is used only for calculating *R*-factors(gt) *etc*. and is not relevant to the choice of reflections for refinement. *R*-factors based on *F*^2^ are statistically about twice as large as those based on *F*, and *R*- factors based on ALL data will be even larger.

### Fractional atomic coordinates and isotropic or equivalent isotropic displacement parameters (Å^2^)

**Table:**

	*x*	*y*	*z*	*U*_iso_*/*U*_eq_	
Br1	0.01364 (4)	0.64928 (2)	0.325242 (18)	0.01732 (7)	
Br3	0.63722 (4)	0.41938 (2)	0.166955 (18)	0.01767 (7)	
F3	0.2805 (3)	0.01545 (14)	0.61742 (12)	0.0256 (3)	
F4	−0.1808 (3)	0.97463 (15)	−0.24671 (12)	0.0269 (3)	
O1	0.4729 (3)	0.76521 (17)	0.16467 (14)	0.0197 (3)	
C1	0.1701 (4)	0.1693 (2)	0.44729 (19)	0.0198 (5)	
H1	0.0491	0.1177	0.4421	0.024*	
C2	0.3155 (4)	0.1313 (2)	0.53742 (19)	0.0177 (5)	
C3	0.4942 (4)	0.2065 (2)	0.5500 (2)	0.0199 (5)	
H3	0.5871	0.1797	0.6124	0.024*	
C4	0.5301 (4)	0.3239 (2)	0.46579 (19)	0.0189 (5)	
H4	0.6503	0.3759	0.4716	0.023*	
C5	0.3885 (4)	0.3646 (2)	0.37295 (18)	0.0157 (4)	
C6	0.2097 (4)	0.2867 (2)	0.36490 (19)	0.0188 (5)	
H6	0.1147	0.3136	0.3031	0.023*	
C7	0.4417 (4)	0.4886 (2)	0.28267 (18)	0.0152 (4)	
H7	0.5313	0.5473	0.3131	0.018*	
C24	−0.1493 (4)	0.7950 (2)	−0.08429 (19)	0.0180 (4)	
H24	−0.2841	0.7630	−0.1019	0.022*	
C25	−0.0292 (4)	0.7287 (2)	0.01293 (18)	0.0150 (4)	
H25	−0.0851	0.6520	0.0613	0.018*	
C26	0.1738 (4)	0.7767 (2)	0.03802 (18)	0.0133 (4)	
C27	0.2560 (4)	0.8922 (2)	−0.03527 (18)	0.0146 (4)	
H27	0.3918	0.9243	−0.0188	0.018*	
C28	0.1387 (4)	0.9599 (2)	−0.13229 (19)	0.0178 (5)	
H28	0.1938	1.0366	−0.1811	0.021*	
C29	−0.0623 (4)	0.9097 (2)	−0.15370 (18)	0.0176 (5)	
C30	0.3079 (4)	0.7134 (2)	0.14074 (18)	0.0146 (4)	
C31	0.2346 (4)	0.5802 (2)	0.22021 (18)	0.0155 (4)	
H31	0.1558	0.5266	0.1802	0.019*	

### Atomic displacement parameters (Å^2^)

**Table:**

	*U*^11^	*U*^22^	*U*^33^	*U*^12^	*U*^13^	*U*^23^
Br1	0.01593 (12)	0.01512 (11)	0.02036 (13)	0.00004 (8)	0.00191 (8)	−0.00485 (8)
Br3	0.01936 (12)	0.01393 (11)	0.02088 (13)	−0.00394 (8)	0.00569 (9)	−0.00655 (8)
F3	0.0373 (9)	0.0182 (7)	0.0176 (7)	−0.0072 (6)	0.0014 (6)	0.0037 (5)
F4	0.0366 (9)	0.0234 (7)	0.0175 (7)	0.0027 (6)	−0.0117 (6)	−0.0004 (6)
O1	0.0211 (8)	0.0162 (7)	0.0228 (9)	−0.0080 (6)	−0.0057 (7)	−0.0027 (6)
C1	0.0231 (12)	0.0161 (10)	0.0205 (12)	−0.0076 (9)	0.0003 (9)	−0.0027 (9)
C2	0.0228 (12)	0.0128 (10)	0.0153 (10)	−0.0001 (8)	0.0022 (8)	−0.0009 (8)
C3	0.0228 (12)	0.0180 (11)	0.0171 (11)	0.0004 (9)	−0.0052 (9)	−0.0015 (9)
C4	0.0208 (11)	0.0172 (10)	0.0193 (11)	−0.0033 (8)	−0.0035 (9)	−0.0048 (9)
C5	0.0194 (11)	0.0122 (9)	0.0151 (10)	−0.0018 (8)	−0.0002 (8)	−0.0026 (8)
C6	0.0220 (11)	0.0165 (10)	0.0168 (11)	−0.0048 (8)	−0.0046 (9)	−0.0004 (8)
C7	0.0159 (10)	0.0138 (9)	0.0170 (11)	−0.0023 (8)	−0.0003 (8)	−0.0057 (8)
C24	0.0165 (11)	0.0177 (10)	0.0213 (12)	−0.0017 (8)	−0.0026 (9)	−0.0074 (9)
C25	0.0171 (10)	0.0132 (9)	0.0150 (10)	−0.0036 (8)	0.0010 (8)	−0.0035 (8)
C26	0.0166 (10)	0.0099 (9)	0.0134 (10)	−0.0002 (7)	−0.0001 (8)	−0.0036 (7)
C27	0.0164 (10)	0.0107 (9)	0.0181 (11)	−0.0026 (8)	0.0029 (8)	−0.0061 (8)
C28	0.0257 (12)	0.0111 (9)	0.0150 (11)	−0.0011 (8)	0.0018 (9)	−0.0013 (8)
C29	0.0234 (11)	0.0145 (10)	0.0134 (10)	0.0061 (8)	−0.0028 (8)	−0.0046 (8)
C30	0.0170 (10)	0.0110 (9)	0.0163 (10)	−0.0030 (8)	−0.0011 (8)	−0.0036 (8)
C31	0.0188 (11)	0.0127 (9)	0.0151 (10)	−0.0041 (8)	−0.0019 (8)	−0.0022 (8)

### Geometric parameters (Å, °)

**Table:**

Br1—C31	1.974 (2)	C7—C31	1.511 (3)
Br3—C7	2.001 (2)	C7—H7	0.9800
F3—C2	1.352 (2)	C24—C29	1.383 (3)
F4—C29	1.347 (2)	C24—C25	1.395 (3)
O1—C30	1.218 (3)	C24—H24	0.9300
C1—C2	1.380 (3)	C25—C26	1.392 (3)
C1—C6	1.384 (3)	C25—H25	0.9300
C1—H1	0.9300	C26—C27	1.398 (3)
C2—C3	1.383 (3)	C26—C30	1.484 (3)
C3—C4	1.392 (3)	C27—C28	1.389 (3)
C3—H3	0.9300	C27—H27	0.9300
C4—C5	1.394 (3)	C28—C29	1.378 (3)
C4—H4	0.9300	C28—H28	0.9300
C5—C6	1.386 (3)	C30—C31	1.537 (3)
C5—C7	1.502 (3)	C31—H31	0.9800
C6—H6	0.9300		
			
C2—C1—C6	118.1 (2)	C25—C24—H24	120.9
C2—C1—H1	121.0	C24—C25—C26	120.4 (2)
C6—C1—H1	121.0	C24—C25—H25	119.8
F3—C2—C1	118.4 (2)	C26—C25—H25	119.8
F3—C2—C3	118.5 (2)	C25—C26—C27	119.3 (2)
C1—C2—C3	123.0 (2)	C25—C26—C30	123.47 (19)
C2—C3—C4	117.6 (2)	C27—C26—C30	117.3 (2)
C2—C3—H3	121.2	C28—C27—C26	121.2 (2)
C4—C3—H3	121.2	C28—C27—H27	119.4
C5—C4—C3	120.9 (2)	C26—C27—H27	119.4
C5—C4—H4	119.5	C29—C28—C27	117.7 (2)
C3—C4—H4	119.5	C29—C28—H28	121.1
C6—C5—C4	119.2 (2)	C27—C28—H28	121.1
C6—C5—C7	122.1 (2)	F4—C29—C28	118.8 (2)
C4—C5—C7	118.6 (2)	F4—C29—C24	118.0 (2)
C1—C6—C5	121.1 (2)	C28—C29—C24	123.2 (2)
C1—C6—H6	119.5	O1—C30—C26	121.82 (19)
C5—C6—H6	119.5	O1—C30—C31	118.95 (19)
C5—C7—C31	116.96 (19)	C26—C30—C31	119.22 (19)
C5—C7—Br3	108.97 (15)	C7—C31—C30	111.97 (19)
C31—C7—Br3	103.93 (16)	C7—C31—Br1	108.95 (16)
C5—C7—H7	108.9	C30—C31—Br1	105.03 (14)
C31—C7—H7	108.9	C7—C31—H31	110.3
Br3—C7—H7	108.9	C30—C31—H31	110.3
C29—C24—C25	118.2 (2)	Br1—C31—H31	110.3
C29—C24—H24	120.9		
			
C6—C1—C2—F3	−178.9 (2)	C30—C26—C27—C28	−178.6 (2)
C6—C1—C2—C3	1.5 (4)	C26—C27—C28—C29	0.3 (3)
F3—C2—C3—C4	178.8 (2)	C27—C28—C29—F4	179.2 (2)
C1—C2—C3—C4	−1.5 (4)	C27—C28—C29—C24	−0.9 (4)
C2—C3—C4—C5	0.8 (4)	C25—C24—C29—F4	−179.1 (2)
C3—C4—C5—C6	0.0 (4)	C25—C24—C29—C28	1.1 (4)
C3—C4—C5—C7	−177.5 (2)	C25—C26—C30—O1	−173.5 (2)
C2—C1—C6—C5	−0.6 (4)	C27—C26—C30—O1	5.2 (3)
C4—C5—C6—C1	−0.1 (4)	C25—C26—C30—C31	5.4 (3)
C7—C5—C6—C1	177.4 (2)	C27—C26—C30—C31	−175.9 (2)
C6—C5—C7—C31	36.4 (3)	C5—C7—C31—C30	172.58 (18)
C4—C5—C7—C31	−146.1 (2)	Br3—C7—C31—C30	−67.3 (2)
C6—C5—C7—Br3	−81.0 (2)	C5—C7—C31—Br1	56.8 (2)
C4—C5—C7—Br3	96.5 (2)	Br3—C7—C31—Br1	176.97 (9)
C29—C24—C25—C26	−0.6 (3)	O1—C30—C31—C7	−31.3 (3)
C24—C25—C26—C27	0.0 (3)	C26—C30—C31—C7	149.7 (2)
C24—C25—C26—C30	178.7 (2)	O1—C30—C31—Br1	86.8 (2)
C25—C26—C27—C28	0.1 (3)	C26—C30—C31—Br1	−92.2 (2)

### Table 1 *Y*—*X*···*Cg* interactions (Å)

Cg1 and Cg2 are the centroids of the C1–C6 and C10–C15 rings, respectively.

**Table:**

*Y*—*X*···*Cg*	*X*···*Cg*	*Y*···*Cg*	*Y*—*X*···*Cg*
C8—Br1···Cg1^i^	3.650 (7)	5.617 (2)	174
C7—Br3···Cg2^ii^	3.479 (6)	5.341 (1)	153

Symmetry codes: (i) -x, 1-y, 1-z ; (ii) 1-x, 1-y, -z.

## References

[bb1] Bruker (2008). *APEX2*, *SAINT* and *SADABS* Bruker AXS Inc., Madison, Wisconsin, USA.

[bb2] Butcher, R. J., Yathirajan, H. S., Sarojini, B. K., Narayana, B. & Mithun, A. (2006). *Acta Cryst.* E**62**, o1629–o1630.

[bb3] Indira, J., Karat, P. P. & Sarojini, B. K. (2002). *J. Cryst. Growth*, **242**, 209–214.

[bb4] Narayana, B., Mayekar, A. N., Yathirajan, H. S., Sarojini, B. K. & Kubicki, M. (2007). *Acta Cryst.* E**63**, o4362.

[bb5] Sarojini, B. K., Narayana, B., Yathirajan, H. S., Mayekar, A. N. & Bolte, M. (2007). *Acta Cryst.* E**63**, o3755.

[bb6] Sheldrick, G. M. (2008). *Acta Cryst.* A**64**, 112–122. 10.1107/S0108767307043930 18156677

[bb7] Tam, W., Guerin, B., Calabrese, J. C. & Stevenson, S. H. (1989). *Chem. Phys. Lett.* **154**, 93–96.

[bb8] Uchida, T., Kozawa, K., Sakai, T., Aoki, M., Yoguchi, H., Abduryim, A. & Watanabe, Y. (1998). *Mol. Cryst. Liq. Cryst.* **315**, 135–140.

[bb9] Yathirajan, H. S., Vijesh, A. M., Narayana, B., Sarojini, B. K. & Bolte, M. (2007). *Acta Cryst.* E**63**, o2198–o2199.

[bb10] Zhao, B., Lu, W. Q., Zhou, Z. H. & Wu, Y. (2002). *J. Mater. Chem.* **10**, 1513–1517.

